# Lifetime History of Concussion Among Youth With ADHD Presenting to a Specialty Concussion Clinic

**DOI:** 10.3389/fneur.2021.780278

**Published:** 2022-01-20

**Authors:** Nathan E. Cook, Elizabeth Teel, Grant L. Iverson, Debbie Friedman, Lisa Grilli, Isabelle Gagnon

**Affiliations:** ^1^Department of Physical Medicine and Rehabilitation, Harvard Medical School, Boston, MA, United States; ^2^MassGeneral Hospital for Children Sports Concussion Program, Boston, MA, United States; ^3^Department of Physical Medicine and Rehabilitation, Spaulding Rehabilitation Hospital, Charlestown, MA, United States; ^4^School of Physical and Occupational Therapy, McGill University, Montréal, QC, Canada; ^5^Spaulding Research Institute, Charlestown, MA, United States; ^6^Montreal Children's Hospital, McGill University Health Centre, Montréal, QC, Canada; ^7^Department of Pediatrics and Pediatric Surgery, Faculty of Medicine and Health Sciences, McGill University, Montréal, QC, Canada; ^8^CHIRPP/Public Health Agency of Canada, Montréal, QC, Canada

**Keywords:** children, adolescents, attention-deficit/hyperactivity disorder, mild traumatic brain injury, health history

## Abstract

Child and adolescent student athletes with attention-deficit/hyperactivity disorder (ADHD) report a greater lifetime history of concussion than those without ADHD. This case-control study compared youth with and without ADHD presenting for care at a specialty concussion clinic on their lifetime history of concussion. We hypothesized that a greater proportion of youth with ADHD would report a history of prior concussion. Archival clinical data from patients presenting to a specialty concussion clinic in Montreal, Québec, Canada between September 2015 and August 2019 were analyzed. The sample included 2,418 children and adolescents (age: *M* = 13.6, *SD* = 2.7, range 5–18 years; 50.9% girls), including 294 (12.2%) with ADHD and 2,124 (87.8%) without ADHD. The proportion with prior concussion among youth with ADHD (43.9%) was significantly greater than youth without ADHD [37.5%, χ^2^ = 4.41, *p* = 0.04, *OR* = 1.30, 95% confidence interval (CI): 1.02–1.67]. A significantly higher proportion of boys with ADHD had a prior concussion history (48.1%) than boys without ADHD [38.4%, χ^2^ = 5.33, *p* = 0.02, *OR* = 1.48 (95% CI: 1.06–2.09)], but this difference was not observed for girls (χ^2^ = 0.31, *p* = 0.58). Youth with ADHD did not differ with regard to their estimated longest duration of symptoms from a prior concussion (*Z* = 1.52, *p* = 0.13) and the proportion who reported taking longer than 28 days to recover from a prior concussion did not differ between those with ADHD (15.3%) and without ADHD (12.2%), χ^2^ = 2.20, *p* = 0.14. Among youth presenting to a specialty clinic, ADHD was associated with greater lifetime history of concussion but not a greater duration of symptoms from a prior injury.

## Introduction

Numerous studies have found that children and adolescents with attention-deficit/hyperactivity disorder (ADHD) report a greater lifetime history of concussion than those who do not have ADHD (1–5). For example, among high school student athletes (ages 13–18) surveyed before participating in a sports season, 24.5% of those with self-reported ADHD reported a history of prior concussion, compared to 16.1% of students without ADHD (5). Similarly, among middle school student athletes (ages 11–14) surveyed before participating in a sports season, 23.9% of those with self-reported ADHD reported a history of prior concussion, compared to 11.4% of those without ADHD (3). This finding has been replicated in a large sample of children (*N* = 10,585) from the U.S. general population ages 9 and 10 years old. Namely, the prevalence of prior concussion among children with ADHD was 7.2% compared to 3.2% among children without ADHD, meaning ADHD is associated with twice the odds of experiencing a prior concussion before age 11 (6). Moreover, sex differences have been reported. Among student athletes from the United States, boys with ADHD report a greater lifetime history of concussion than girls with ADHD (3–5).

Studies have primarily examined samples of uninjured student–athletes who complete health history surveys at the beginning of sporting seasons. To our knowledge, no prior study has examined the association between ADHD and lifetime history of concussion among youth who present for clinical care after sustaining a concussion. Several studies of concussion specialty clinic patients have reported lifetime history of concussion in their samples. We extracted the proportions from 16 of these studies and determined, on average (weighted mean proportion), about 35% of youth presenting to concussion clinics report having a prior concussion (7–22), with proportions ranging from 21% (19) to 60% (20) across studies. That said, no prior clinic-based study has stratified concussion history by ADHD status. Further, studies have predominantly examined the association between ADHD and concussion among youth in the United States, with only one study reporting on this association among youth in Canada (1).

Using a large archival dataset, we examined the proportions of youth with and without ADHD who report a lifetime history of concussion when they present for treatment at a Canadian specialty concussion clinic. We hypothesized that (i) youth with ADHD would have a greater lifetime history of concussion than youth without ADHD, and (ii) boys with ADHD would have a greater lifetime history of concussion than girls with ADHD.

## Materials and Methods

### Design and Participants

Participants included concussed children and adolescents who presented for treatment at a specialty concussion clinic in Montreal, Quebec, Canada consecutively between September 2015 and August 2019. The sample included 2,418 children and adolescents (see Table 1). The mean age of the sample was 13.6 years (*SD* = 2.7, range: 5–18) and equally split between girls (50.9%) and boys (49.1%). The primary (i.e., preferred or dominant) language spoken was French (57.8%), English (41.7%), and bilingual/equal fluency in both languages (0.5%). Data regarding racial and ethnic group affiliation were not available. A trauma coordinator, who is a licensed health care professional, prospectively gathered this information during a customary intake interview using standardized data collection forms, with the parent and child collaboratively providing answers. Specifically, youth and their caregivers were asked whether youth had a pre-injury diagnosis of ADHD (yes or no) and also reported the youth's concussion history (yes/at least one prior concussion or no prior concussion). Those with previous concussions estimated the longest duration of symptoms that they experienced from a prior concussion.

**Table 1:** Sample demographics.

	**Total**	**ADHD**	**No ADHD**	
	***N* = 2,418**	***n* = 294**	***n* = 2,124**	***P*-value**
Age at injury, *Median* (IQR)	14 (12–16)	15 (13–16)	14 (12–16)	<0.001
Gender, *n* (%)				
Females	1,230 (50.9)	138 (46.9)	1,092 (51.4)	0.15
Males	1,188 (49.1)	156 (53.1)	1,032 (48.6)	
Primary language, *n* (%)				
French	1,392 (57.8)	205 (70.0)	1,187 (56.1)	<0.001
English	1,004 (41.7)	88 (30.0)	916 (43.3)	
Bilingual	13 (0.5)	0 (0.0)	13 (0.6)	
Academic performance, *n* (%)				
Above average	1,095 (46.8)	74 (26.6)	1,021 (49.5)	<0.001
Average	977 (41.8)	130 (46.8)	847 (41.1)	
Below average	267 (11.4)	74 (26.6)	193 (9.4)	
Mechanism of index injury, *n* (%)				
Sport	1,617 (66.9)	186 (63.2)	1,431 (67.4)	0.16
Non-sport	801 (33.1)	108 (36.7)	693 (32.6)	
Referral source, *n* (%)				
Emergency department	1,350 (55.8)	155 (52.7)	1,195 (56.3)	0.25
Other	1,068 (44.2)	139 (47.3)	929 (43.7)	
History of migraines, *n* (%)				
Yes	1,013(41.9)	160 (54.4)	853 (40.2)	<0.001
No	1,405 (58.1)	134 (45.6)	1,271 (59.8)	
History of sleep disorder, *n* (%)				
Yes	621 (25.7)	146 (49.7)	475 (22.4)	<0.001
No	1,797 (74.3)	148 (50.3)	1,649 (77.6)	
History of anxiety, *n* (%)				
Yes	1,152 (47.6)	217 (73.8)	935 (44.0)	<0.001
No	1,266 (52.4)	77 (26.2)	1,189 (56.0)	
History of depression, *n* (%)				
Yes	275 (11.4)	82 (27.9)	193 (9.1)	<0.001
No	2,143 (88.6)	212 (72.1)	1,931 (90.9)	
History of learning disorder, *n* (%)				
Yes	148 (6.1)	75 (25.5)	73 (3.4)	<0.001
No	2,270 (93.9)	219 (74.5)	2,051 (96.6)	
Days between index injury and presentation to clinic, *Median* (IQR)	15 (8–33)	14 (8–36)	15 (8–33)	0.39

*The mechanism of index injury refers to the youth's most recent injury for which they presented to the clinic for treatment, not the mechanism for any prior lifetime injuries. ADHD, attention-deficit/hyperactivity disorder; P-values are for significance tests comparing youth with and without ADHD*.

### Statistical Analyses

The proportions of youth with and without ADHD who had a history of concussion (one or more prior injuries) were computed and compared using χ^2^-tests. The proportions of girls and boys with ADHD who experienced a prior concussion were also compared using χ^2^-tests. To characterize the magnitude of group differences, an odds ratio (*OR*) was calculated for each analysis as an effect size (23) and interpreted according to widely used criteria (i.e., *OR*s between 1.2 and 1.71 = small, *OR*s between 1.72 and 2.4 = medium, and *OR*s >2.4 = large) (24). Analyses were conducted using SAS (Version 9.4; SAS Institute, Inc., Cary, North Carolina).

## Results

### Descriptive Data

In the total sample, roughly 2 out of 5 (38.3%) concussed youth presenting for care at a specialty concussion clinic had sustained a previous concussion. Boys (39.7%) and girls (37.0%) did not differ with regard to their personal history of concussion, χ^2^ = 1.80, *p* = 0.09, *OR* = 1.12 (95% confidence interval, CI: 0.95–1.32). A total of 294 youth (12.2% of the sample) reported having been diagnosed with ADHD. Additionally, sex differences were not observed amongst children and adolescents with ADHD [Boys: 53.0%, Girls: 47.0%, χ^2^ = 2.07, *p* = 0.15, *OR* = 1.20 (0.94–1.53)]. No overall differences in mechanism of injury or referral source were observed, as 67.4% of youth without ADHD and 63.3% of youth with ADHD were concussed through a sport-related mechanism [χ^2^ = 1.97, *p* = 0.16, *OR* = 1.20 (0.93–1.55)] and 56.2% of youth without ADHD and 52.7% of youth with ADHD were referred to the concussion clinic from the pediatric emergency room compared to other referral sources [χ^2^ = 0.41, *p* = 0.52, *OR* = 1.09 (0.83–1.44)].

### Lifetime History of Concussion

A significantly greater proportion of youth with ADHD [43.9% (95% CI 38.2–49.6%)] reported a prior concussion history compared to youth without ADHD [37.5% (35.5–39.6%)], χ^2^ = 4.41, *p* = 0.04, *OR* = 1.30 (1.02–1.67). The proportion with a prior concussion among boys with ADHD [48.1% (40.2–55.9%)] did not significantly differ from girls with ADHD [39.1% (31.0–47.2%)], χ^2^ = 2.38 *p* = 0.12, *OR* = 1.15 (0.91–2.29). However, the proportion with a prior concussion among boys with ADHD [48.1% (31.6–63.1%)] differed significantly from boys without ADHD [38.4% (35.4–41.3%)], χ^2^ = 5.33, *p* = 0.02, *OR* = 1.49 (1.06–2.09). This finding was not observed among girls [girls with ADHD: 39.1% (31.0–47.3%); girls without ADHD: 36.7% (33.9–39.6%); χ^2^ = 0.31 *p* = 0.58, *OR* = 1.11 (0.77–1.59)].

We also examined lifetime history of concussion among youth with and without ADHD stratified by the mechanism of their index injury, i.e., their most recent injury, for which they were receiving care (sport-related index injury vs. non-sporting injury), as well as stratified by the referral source for their index injury (emergency room vs. another source). Of those youth presenting for care following a sport-related concussion, the proportion of lifetime history of prior concussion did not differ in youth with ADHD [46.2% (39.1–53.4%)] compared to youth without ADHD [40.1% (37.6–42.7%)], χ^2^ = 2.56, *p* = 0.11, *OR* = 1.28 (0.94–1.75). Of those youth receiving care for a non-sporting injury, the proportion of lifetime history of prior concussion did not differ in youth with ADHD [39.8% (30.6–49.1%)] compared to youth without ADHD [32.2% (28.7–35.7%)], χ^2^ = 2.46, *p* = 0.12, *OR* = 1.39 (0.92–2.12). For individuals referred from the pediatric emergency department, the proportion of prior concussion significantly differed in youth with ADHD [47.7% (39.9–55.6%)] compared to youth without ADHD [38.0% (35.2–40.7%)], χ^2^ = 5.48, *p* = 0.02, *OR* = 1.49 (1.07–2.09). For individuals referred from sources other than a pediatric emergency department, the proportion of prior concussion did not differ in youth with ADHD [39.6% (31.4–47.7%)] compared to youth without ADHD [36.9% (33.8–40.0%)], χ^2^ = 0.36, *p* = 0.55, *OR* = 1.12 (0.78–1.61).

### Longest Symptom Duration From a Previous Concussion

Among all children and adolescents who reported a prior history of concussion (*n* = 702), the median longest symptom duration from a previous injury was 21 days (IQR = 10–42 days; range 1–480). Youth with ADHD (median = 21 days, IQR: 14–60) did not differ from youth without ADHD (median = 21 days, IQR: 10–40) with regard to longest duration of symptoms from a prior concussion, *Z* = 1.52, *p* = 0.13. Further, the proportion of youth who reported taking longer than 28 days to recover from a prior concussion did not differ between those with ADHD (15.3%) and without ADHD (12.2%), χ^2^ = 2.20, *p* = 0.14.

## Discussion

There is a body of literature indicating that children (3), adolescents (4, 5), and young adults (25) with ADHD report a greater lifetime history of concussion than youth who do not have ADHD. Moreover, the majority of studies report a sex difference, such that boys report a greater history of concussion than girls, and boys with ADHD report a greater lifetime history of concussion than girls with ADHD (3–5). The results from the present study are consistent with previously published studies, and support our hypothesis that youth with ADHD who presented for care in a specialty concussion clinic report a greater lifetime history of concussion compared to those without ADHD. Specifically, 43.9% of youth with ADHD had experienced a prior concussion, compared to 37.5% of youth without ADHD. Additionally, we found a significant sex difference, though not the hypothesized sex difference. Namely, 48.1% of boys with ADHD reported a prior concussion history compared to only 38.4% of boys without ADHD. Boys with ADHD did not significantly differ from girls with ADHD with regard to proportions with prior concussion and girls with ADHD did not differ from girls without ADHD with regard to proportions with prior concussion. Finally, among youth referred for care from an emergency department, ADHD was associated with disproportionate history of previous concussion.

The consistency between the current results and prior studies is especially interesting given notable differences in the populations being sampled and the research methodology used. The current sample of youth from the community who are presenting for clinical care at a specialty clinic differ in several ways from child and adolescent student athletes who complete health history surveys during preseason baseline assessments. For example, rates of ADHD and rates of prior concussion are notably higher in the current sample compared to previous studies of student athletes completing pre-participation health surveys. Specifically, ADHD was overrepresented in the current clinical sample, with 1 in 10 patients (12.2%) reporting a prior diagnosis of ADHD compared to about 6% in student athlete studies (3, 4). Similarly, the proportion of those with prior concussion was considerably higher in the current study, with nearly two of five patients (38.3%) having sustained a prior concussion, compared to 12% of middle school student athletes (3) and 20% of high school student athletes (4, 5). Despite the high proportions of those with prior concussion among this sample of youth from the community seeking healthcare, the ADHD association was still present. There are also differences in data collection methods to consider, as studies of student athletes examine child or adolescent self-reported concussion history on self-guided surveys, whereas in the current study, caregivers were with youth to provide health history during the course of an intake interview with a clinician, who asks specific and direct questions—which might make the health history information more accurate. Moreover, it is important to highlight that the current study sampled *injured* youth from the community presenting for healthcare, as opposed to *uninjured* student athletes, and further, these injured youth presented to clinic, and provided their health history information for this study, a median of 15 days post injury. Children and adolescents often experience rapid symptom improvement and resolution within the first 14 days following concussion (26), and the majority of children pursuing care in this clinic are on a trajectory for experiencing slow recovery. Thus, among this clinical population of community youth seeking healthcare who are, on average, experiencing slow recovery, the association between ADHD and prior history of concussion was once again observed.

**Figure 1:**
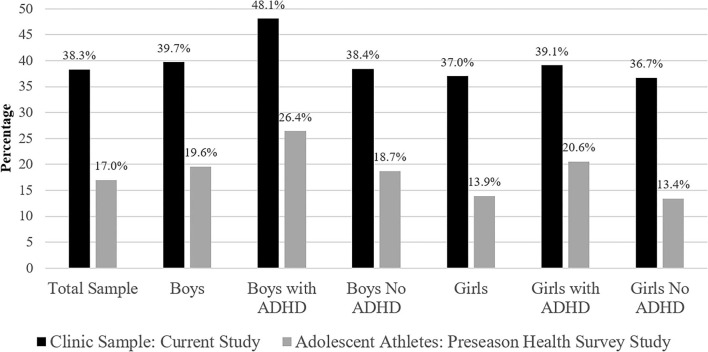
Percentages of youth reporting a prior personal history of concussion. Sample sizes for the study of youth completing a preseason health survey were as follows: Total = 32,487, Boys = 14,367, Boys with ADHD = 907, Boys No ADHD = 13,460, Girls = 13,338, Girls with ADHD = 417, Girls No ADHD = 12,921 (5).

As seen in Figure 1, the results from this study are compared to a prior study of more than 32,000 adolescents from the state of Maine who completed a health survey prior to competing in sports. The most striking difference is the much higher rate of prior concussion in the specialty clinic sample. The proportion of youth with prior concussion from the current clinic sample (38.3%) is larger than the student athlete study (17%) (5), but is highly consistent with results from numerous clinic-based studies that have reported approximately 35% of youth presenting to concussion clinics have a prior concussion history (i.e., based on 16 studies, the weighted mean proportion was 34.5% and the range was 21.0–60.3%) (7–22). Moreover, in one pediatric primary care sample, the proportion of concussed youth with a prior concussion history (26.7%) (27) was greater than student athletes surveyed during baseline assessments and lower than injured youth presenting to most specialty clinics. Higher proportions of previous concussion among youth presenting to specialty clinics might reflect, in part, that youth (28) and young adult (29) athletes with a past history of concussion are at increased risk for a future concussion. It also seems likely that families of children and adolescents with a past history of concussion might be more likely to seek out and attend a specialty concussion clinic following their next injury.

This study is novel in that it is the first to compare the estimated longest symptom duration from previous concussions between youth with and without ADHD. Despite consistent findings that youth with ADHD experience greater lifetime concussion histories, youth with ADHD did not differ from youth without ADHD with regard to longest duration of symptoms from a prior concussion (median for both groups was 21 days). Similarly, the proportion of children and adolescents who reported taking longer than 28 days to recover (commonly considered the demarcation point for “persistent” or prolonged recover) from a prior concussion did not differ between those with ADHD (15.3%) and without ADHD (12.2%). This novel result aligns with conclusions from systematic reviews (30, 31) as well as recent prospective evidence (32) that ADHD does not appear to be a risk factor for prolonged symptoms or worse outcome following concussion.

## Limitations

This study has several methodological limitations. First, both ADHD status and concussion history were reported by the parents and youth; neither were verified using a clinical evaluation or by reviewing medical records. With that said, caregivers were present with the youth and this health history information was collected during a clinical interview with a trauma coordinator, and thus likely represents a more valid data collection process than many studies on this topic, which rely exclusively on unaided youth self-report of health history. Second, we do not know the subtype or severity of ADHD, age of ADHD onset, current ADHD symptom burden, or whether the youth were on medications for ADHD. Third, we did not have access to racial or ethnic group identification, and more refined examination and understanding of sociocultural factors in concussion research is an important future direction for the field. That said, this study examined Canadian youth whose predominant preferred or dominant language was French, a clinical population that, to this point, has not been characterized with respect to potential associations between pre-existing ADHD and lifetime concussion history. Finally, we do not know how many prior concussions the children and adolescents experienced (the data were coded as present/absent), how long ago the concussion(s) occurred, or the severity of those injuries.

## Conclusion

Among samples of student athletes and children from the U.S. general population, ADHD is consistently associated with greater lifetime history of concussion. This finding is consistent among the current sample of youth who presented for care at a specialty clinic in Canada, such that among children and adolescents who are seeking health care for an index concussion, those with ADHD report greater lifetime history of concussion than youth without ADHD. However, youth with ADHD did not differ from youth without ADHD in their estimated longest duration of symptoms from a prior concussion—consistent with past studies suggesting that ADHD, per se, does not appear to be a risk factor for prolonged symptoms following concussion.

## Data Availability Statement

The original contributions presented in the study are included in the article. Further inquiries can be directed to the corresponding author. The statistical code, syntax, output, analyses, and minimum dataset are available to qualified researchers upon request.

## Ethics Statement

The studies involving human participants were reviewed and approved by Ethics approval was provided by the McGill University Health Centre (Pediatrics Review Board; #2019-4723). Written informed consent from the participants' legal guardian/next of kin was not required to participate in this study in accordance with the national legislation and the institutional requirements.

## Author Contributions

NC helped conceptualize the study, conceptualized the statistical analyses, drafted sections of the manuscript, assisted with the literature review, edited the manuscript, and approved the final manuscript. ET helped design and coordinate data collection, conducted the statistical analyses, drafted sections of the manuscript, critically reviewed, and edited/approved the final manuscript. GI helped conceptualize the study, assisted with the literature review, drafted sections of the manuscript, edited the manuscript, and approved the final manuscript. DF and LG critically reviewed and edited/approved the final manuscript. IG helped design and coordinate data collection, wrote the IRB, conceptualized the overall project, critically reviewed, and edited/approved the final manuscript. All authors approved the final manuscript as submitted and agree to be accountable for all aspects of the work.

## Funding

NC acknowledges support from the Louis V. Gerstner III Research Scholar Award from Massachusetts General Hospital. GI acknowledges unrestricted philanthropic support from the Mooney-Reed Charitable Foundation, Heinz Family Foundation, Boston Bolts, ImPACT^®^ Applications, Inc., National Rugby League, and the Spaulding Research Institute.

## Conflict of Interest

GI has a clinical practice in forensic neuropsychology, including expert testimony, involving individuals who have sustained mild TBIs. He has received research support from the Harvard Integrated Program to Protect and Improve the Health of NFLPA Members, and a grant from the National Football League. He serves as a scientific advisor for NanoDx™, Sway Medical, Inc., and Highmark, Inc. The remaining authors declare that the research was conducted in the absence of any commercial or financial relationships that could be construed as a potential conflict of interest.

## Publisher's Note

All claims expressed in this article are solely those of the authors and do not necessarily represent those of their affiliated organizations, or those of the publisher, the editors and the reviewers. Any product that may be evaluated in this article, or claim that may be made by its manufacturer, is not guaranteed or endorsed by the publisher.
